# Research on the Structural Relationship of Online Persistent Purchase of Museum Cultural and Creative Products in the Context of Digitalization

**DOI:** 10.3389/fpsyg.2022.881957

**Published:** 2022-04-13

**Authors:** Mengyi Lin, Zhaoyang Meng, Caisheng Luo

**Affiliations:** College of Design and Innovation, Fujian Jiangxia University, Fuzhou, China

**Keywords:** museum cultural and creative products, perceived interest, perceived value, involvement in purchasing decisions, persistent purchase

## Abstract

With the development and support of modern technologies such as digital media and online live stream, it has become an effective way to promote the online transaction of museum cultural and creative products. Based on the Technology Acceptance Model combined with the Post-Acceptance Model of Information System Continuance and the theories on customer satisfaction index, this research introduces perceived interest (PI) and media richness (MR) as variables and constructs a model of the influencing factors of online consumers’ persistent purchase (PP) of museum products. The research model and related hypotheses were verified through structural equation modeling (SEM). The research found that perceived value (PV) and satisfaction (SAT) are the key variables that have impact on the PP of consumers. Perceived quality, brand trust, MR, PI, and perceived usefulness have significant effects on PV and SAT. Involvement in purchasing decisions positively moderates the impact on SAT of PV. The conclusion can be referred to for operators and product developers.

## Introduction

Since 2019, the museum cultural and creative industry has been integrated into the digital economy, building a cross-border cultural and creative industry chain. Many Internet companies have been deeply involved in the museum cultural and creative industry, comprehensively participating in all aspects of the cultural and creative industry chain such as user survey, product design, and promotion and marketing. The emerging Internet technology, digital media, virtual reality, interaction design theory, etc., have been gradually applied to the practice of museum operation, greatly improving the digital, networked and intelligent development level of museums and promoting the interconnection of people, objects and data in the museum (Zhang and Zhang, 2017). Multi-dimensional communication and diverse presentation methods also provide new ideas and methods for the popularization of cultural and creative products. Especially during the COVID-19 pandemic, e-services provide opportunities for tourists (Gutowski and Kłos-Adamkiewicz, 2020).

Museum exhibitions and activities are transforming from offline to online, and several online platforms have even started live sales. Museums have become increasingly focused on using their websites to communicate with their audiences (King et al., 2021). According to the 2021 museum data report of China’s TikTok (a China’s short video social APP with more than 600 million daily active users), by May 2021, the number of museum-related videos on TikTok, the short video platform, has exceeded 33.89 million, and such videos have been played more than 72.3 billion times and received more than 2.1 billion likes (Tik Tok, 2021), which demonstrates the power of digital platforms to expand the experience in cultural product shopping beyond physical museums. Live stream not only expands the influence of museums, but also provides new marketing channels for museum cultural and creative products. Using short video platforms for popularization has also become an important way for museums to seek economic benefits. However, at the same time, such highly competitive environment also calls for museums’ a large number of efforts to attract visitors and gain popularity so as to increase the number of consumers and the amount of revenue. Coupled with furious competition from other e-commerce platforms, market competition in the cultural and creative industry has been intensified, for which it is necessary to encourage a constant re-examination of cultural and creative product innovation, online store design and its impact on consumer behaviors.

In recent years, scholars have carried out research on museum-related operations from different perspectives. For example, Han et al. (2019) discussed the role of the internal and external environment of museums on visitor experience and loyalty, Tu et al. (2019) conducted the consumer preference research on cultural and creative products, and Wu et al. (2021) constructed a theoretical model of the influencing factors of users’ continuous intentions for digital museums. However, few scholars have explored the development path of consumers’ online repurchase of museum products in the digital age from the perspective of consumers, conducted research on the persistent purchase (PP) of online museum users, or discussed the formation mechanism. Bhattacherjee (2001) pointed out that for information systems, acceptance is only the first step to success, and the ultimate success depends more on the continuous use of users. Similar to other information systems, good user experience and continuous use of users are the keys to the ultimate success of an online museum, especially the PP of customers after the online store’s live stream. However, the competition in the online market is becoming more and more fierce. Even if museums make great efforts, it is difficult for them to retain users, thus affecting economic benefits. In other words, this research proposes that the techniques provided by online stores and online live stream platforms, as well as the behavior of online sellers, will affect the purchase and repurchase intentions of online customers. Therefore, in order to stand out from the competition, museums must know how to intensify the perceived value (PV) of their products and services more appropriately and visibly to increase customer repurchase.

In view of this, research on the influencing factors of online consumers’ PP has important guiding and reference significance for promoting the innovation and operation of online museum cultural and creative products. First of all, this research resorts to the Technology Acceptance Model (TAM) combined with the Post-Acceptance Model of Information System Continuance and the theories on customer satisfaction index, and introduces relevant variables and their basic concepts to discuss the relationship between factors of consumer purchase and their PP in museum online stores. Then, a research model is built to testify the positive effect of relevant factors on consumers’ PP, and the model is empirically tested with data collected from questionnaires filled by consumers who have participated in museum live stream or visited the museums’ online stores. Last but not least, this research discusses how the empirical results help managers, technicians, and researchers further improve the presentation of the online museum store from information presentation to value perception and from transaction process to second dissemination from the perspective of “consumer-oriented” service, to rethink and reanalyze more aspects of museum cultural and creative products such as product development and product marketing from the perspective of customer perception and customer experience, and to form a strategic direction and implementation plan with certain referential value in the field of the regeneration and the industrial ecosystem of cultural and creative products.

This research consists of the following sections to present a theoretical review of relevant literature and the development of hypotheses. Research methods are introduced in section “Research Design and Methods,” data analysis is presented in Results, and empirical results are shown in section “Results and Discussion.” Conclusions, recommendations, and limitations are given in section “Research Conclusions and Recommendations,” followed by references at the end.

## Literature Review and Research Hypotheses

This research adopts the TAM proposed by Davis as the research basis which is mostly used by scholars to analyze user acceptance of various information systems (Davis, 1989; Hu et al., 2021). This theory has been revised and improved many times by scholars, and various TAM extension models have been put forward in different empirical studies. Post-Acceptance Model of Information System Continuance was proposed by Bhattacherjee (2001) and partially adjusted by the expectation confirmation theory, which focuses on the expectation after use and adds “perceived usefulness” to the theory. The main purpose is to predict and explain users’ continuous use of the information system, and to analyze user satisfaction and intention of the continuous use of the information system. The customer satisfaction index model has been improved by scholars for many times, and the four current models with relatively complete system and good application effect are Swedish Customer Satisfaction Barometer (SCSB), American Customer Satisfaction Index (ACSI), European Customer Satisfaction Index (ECSI), and China Customer Satisfaction Index (CCSI) (Cui et al., 2019).

This research believes that the continuous use of information system has many similar variables with the repurchase behavior in the customer satisfaction model, such as customers’ perceived quality (PQ), PV, perceived usefulness (PU), and satisfaction (SAT). Whether the consumers are satisfied with the product or service is judged by comparing their pre-consumption PV and post-use performance, and their SAT provides a reference for repurchase or reuse in the future. Referring to the above theories, related variables including perceived interest (PI) and media richness (MR) are added to expand the research on the post-acceptance PP intention and behavior of online museum consumers in the digital age.

### Persistent Purchase and Satisfaction

Satisfaction has long been considered an important variable in the marketing field to predict behavior and attitudes, as it can assess customer needs or expectations and predict post-use evaluations. SAT is a psychological state, which refers to the subjective evaluation after the generation of consumers’ actual feelings of buying museum cultural and creative products online. PP refers to the degree of subjective willingness of users to continue to use the online museum platform to purchase after initial adoption. In the field of online marketing, users’ PP is affected by many factors, and SAT is regarded as a key factor that positively affects the PP in online stores. When Cardozo (1965) first introduced the concept of SAT to marketing, he proposed that customer SAT will lead to repurchase intentions of customers. Later research and studies by many scholars have also confirmed that customer SAT positively affects users’ PP and feedback (Carmen and Pop, 2012; Yim et al., 2012; Prakriti, 2021). In this research, SAT is used to evaluate and explain users’ willingness to continue to use online museum stores, that is, when users are satisfied with the online museum store, they are more willing to repurchase otherwise, they are likely to stop browsing and switch to other competing products. Therefore, this research proposes the following hypothesis:

H1: Satisfaction positively affects the persistent purchase of online museum consumers.

### Perceived Value

Consumer PV is referred as the user’s evaluation on a certain product or service (Zeithaml, 1988). For online shopping, PV can affect the user’s purchase intention (Chen, 2008; Liu et al., 2021), and is a key antecedent of purchase behavior (Chiu et al., 2014). Li et al. (2007) found that PV and customer SAT are positively related to repurchase intention. Meanwhile, PV is positively related to customer SAT. Zhou and Yu (2021) found that the user’s PV of archival cultural and creative products is closely related to customer SAT, and PV is considered as a direct factor that affects consumer SAT (Jiang, 2013). Previous research and studies by many scholars have also proved that PV determines customer SAT (Bai and Liao, 2001) and has a positive impact on SAT (Ihtiyar et al., 2019). Therefore, the degree of PV can directly influence customer SAT, it is also a crucial element to determine consumers’ PP (Jiang, 2013; Seo and Lee, 2021) and a good predictor to explain their repurchase intention. Based on this, the following hypotheses are proposed:

H2: Perceived value positively impacts on the persistent purchase of online museum consumers.
H3: Perceived value positively impacts on the satisfaction of online museum consumers.

### Involvement in Purchasing Decisions

Oliver (1980) defined involvement as “the degree of attention to specific products and services, which is mainly manifested in paying more attention to consumption, preparing before making purchasing decisions, and better processing consumption results.” The higher the degree of involvement is, the more information consumers collect for making purchasing decisions, and it takes longer and they are more motivated to make satisfying decisions (Oliver and DeSarbo, 1988). Browne and Kaldenberg (1997) proposed that consumers’ behavior of information seeking and evaluation positively affect product involvement and also consumers’ PV. The degree of involvement in purchasing decisions (IPD) mainly reflects the importance that consumers attach to products through their own feelings, which will have a certain impact on consumers’ PQ of products, that is, the degree of IPD will affect consumers’ choice. Yang (2006) believes that the higher the correlation degree between consumers’ involvement and their personal value, needs, and interests, the higher the degree of involvement is. It is shown that higher engagement in purchasing decisions also leads to higher SAT (Green and Chalip, 1998). Therefore, customers of high-degree involvement can make decisions that better meet their needs, which will lead to higher degree of SAT (Oliver and DeSarbo, 1988). In this research, based on the special historical and cultural attributes of museums and consumers’ curiosity about historical relics, more attention that is different from other product attributes can be drawn through the immersion and interactivity of live stream platforms and digital museum. Therefore, the degree of consumers’ IPD will play a significant role in customer SAT formation. Based on this, this research proposes the following hypothesis:

H4: The involvement in purchasing decisions of online museum consumers positively moderates the impact of perceived quality on satisfaction.

### Perceived Quality

Perceived quality is significantly related to consumers’ purchase. Monroe and Krishnan (1985) believe that PQ directly affects PV, which further affects purchase intention. Zeithaml (1988) defined PQ as “consumers’ judgment on the overall excellence or superiority of a product,” and found that the higher consumers’ PQ of a product is, the higher the product’s PV is. Garretson and Clow’s (1999) research also pointed out that consumers’ PQ will affect their willingness to buy a certain product. Previous research shows that PQ is an important predictor of consumers’ purchasing decisions. For example, PQ can positively affect SAT and PV (Beneke et al., 2013; Han and Hyun, 2017). Based on the above research results, the following hypotheses are proposed:

H5: Perceived quality positively impacts on the perceived value of museum online consumers.
H6: Perceived quality positively impacts on the satisfaction of museum online consumers.

### Brand Trust

Brand trust (BT) is customers’ confidence expectations in brand reliability and brand behavioral intentions when facing risks (Munuera-Aleman et al., 2003). Shah and Alter (2014) believe that consumer SAT is related to BT, and that BT is directly affected by SAT. When consumers have higher degree of BT in a product, their PV will be higher. When consumers are willing to start trusting a brand or the products provided by the brand, their confidence in the product will be enhanced, and they are likely to believe it is a good bargain at the most reasonable price, thereby increasing the SAT. Therefore, BT is able to stimulate future behavioral intentions to the brand including repurchase, premium purchase and brand loyalty (Sirdeshmukh et al., 2002; Nguyen and Pervan, 2020). Lin and Lu (2010) proposed that trust significantly affects repurchase intention. Therefore, BT is also a key factor affecting consumers’ intention to repurchase. Nowadays, the power of brands is self-evident, and museums are considered as the heritage of human history and culture, especially for its special official background and cultural soft power. This research believes that BT can become a firm support for the innovation and development of museums, therefore we expect that trust in museum brands will increase online consumers’ PV and SAT. Based on this, the following hypotheses are proposed:

H7: Brand trust positively impacts on the perceived value of online museum consumers.
H8: Brand trust positively impacts on the satisfaction of online museum consumers.

### Media Richness

Media richness refers to the ability of the media to produce information within a specific time interval that can change the recipient’s understanding of information (Daft and Lengel, 1984), it is a website characteristic that provides complete and useful information exchange to better facilitate consumer online interactions by using an effective combination of visual and textual fluency. The evaluation criteria include feedback capacity, multiple cues, channels used, language variety, and personalization (Daft and Lengel, 1984; Levy and Gvili, 2015). For example, through the online museum platform, consumers can browse multiple cues such as sound, animation, graphics, video, and virtual reality provided by the website of museum’s products, thereby enhancing their understanding. Past research and studies have shown that information richness to some extent affects users’ behavior of and decisions on shopping in virtual stores (Chen and Tan, 2004), communication media choices (Lo and Lie, 2008), and digital marketing channels (Levy and Gvili, 2015). Therefore, higher degree of MR will make information easier to understand (Daft and Lengel, 1984), which is a key factor for consumers to understand the details of cultural and creative products in online museum stores. According to the research of Vickery et al. (2004), high-richness media provide guarantee for timely and effective feedback of customer communication and supply network. It can also be said that MR affects user behavior. This research defines MR as allowing consumers to perceive rich and diverse information through multimedia presentation (e.g., images, links, and videos) on the online museum platform. The online museum stores need to be user-oriented and provide interactive feedback. Good interactivity can promote users’ understanding and simplify the process of making purchasing decisions. Displaying cultural and creative products through effective media from multiple perspectives may increase the attractiveness and expectations of users, thereby increasing their SAT. Based on this, it is reasonable to predict that if museums adopt rich media forms, it will potentially improve the PV and SAT of online users. Therefore, this research puts forward the following hypotheses:

H9: Media richness positively impacts on the perceived value of online museum consumers.
H10: Media richness positively impacts on the satisfaction of online museum consumers.

### Perceived Interest

Barnett (1990) in the research of human behavior pointed out that interest mainly focuses on two levels, namely the characteristics and the state of interest. Interest can be defined as the extent to which a user considers the activity of using a product or service to be entertaining, so an external variable, PI is introduced to TAM (Wu et al., 2021). Relevant literature has demonstrated that PI, as an intrinsic motivation, can be regarded as an explanation for the effect of PI in a certain technology or activity on the user intention. For example, Lin et al. (2005) found that PI has a significantly positive impact on the persistent using of website users and on consumer SAT (Hsu et al., 2012). Hu et al. (2011) verified the influence of PI on consumer SAT in the research on consumers’ intention of online shopping. Referring to the theory of User Acceptance Model, Li and He (2020) found that PI can bring joyfulness and enjoyment to consumers, and the diversity of the product itself will bring consumers better experience, which leads to higher evaluation on PV. The conclusion is put forward as PI can positively affect PV and consumers’ purchase intention. Many museums have recently undergone enormous changes in their digital exhibition and social interaction programs, which highlights the potential of enhanced playfulness and uniqueness (Chen et al., 2021). The interest of museum cultural and creative products refers to whether the products bring new and interesting subjective feelings to consumers in terms of visual, tactile, and emotional experience. The higher the degree of product innovation is, the stronger the sense of novelty it brings to consumers. Such subjective PI will improve consumers’ impression on new products, thereby enhancing their purchasing intention.

In this research, the PI of online museum store users is mainly divided into two parts. One is from the perspective of user experience, which reflects users’ subjective evaluation on the degree of concentration and psychological pleasure when browsing the museum cultural and creative products through the online platform, and the other refers to the result of the product interest and pleasure experience that users receive after purchasing. When users gain the enjoyment and joyfulness of browsing the online museum store or post-purchase experience, there will be more positive emotional output, and higher degree of SAT and repurchase intention. Therefore, the hypotheses are proposed as follows:

H11: Perceived interest positively impacts on the perceived value of online museum consumers.
H12: Perceived interest positively impacts on the satisfaction of online museum consumers.

### Perceived Usefulness

The TAM was proposed in the 1980s based on social psychological theory and researches on the relationship between cognitive, affective factors, and technology application. This model has been widely used in the field of information technology (Su and Li, 2021). As one of the main determinants of the TAM, PU has been widely studied, reflecting the degree of improvement in job performance that users subjectively believe when using a particular system. Yao and Qin found through empirical evidence that PU has an important impact on online purchase SAT (Yao and Tan, 2010). Barnes’ research also confirmed that users’ continuous use is closely related to their PU and SAT (Barnes and Böhringer, 2011). Ji’s research on online group purchase proved that usefulness has a direct and positive impact on consumers’ PV, and a direct impact on consumer SAT (Ji, 2015; Yu et al., 2021). For online museum users, PU depends on the resources of online stores, the way they are displayed, and how well the system functions fit the users’ own needs. Online museum product display and interaction can effectively help users improve their decisions. If users consider the information provided on the website about cultural and creative products valuable, they will generate positive emotions which directly affect their purchase intentions. Therefore, the following hypotheses are proposed:

H13: Perceived usefulness positively impacts on the perceived value of online museum consumers.
H14: Perceived usefulness positively impacts on the satisfaction of online museum consumers.

### Proposed Theoretical Model

**FIGURE 1:**
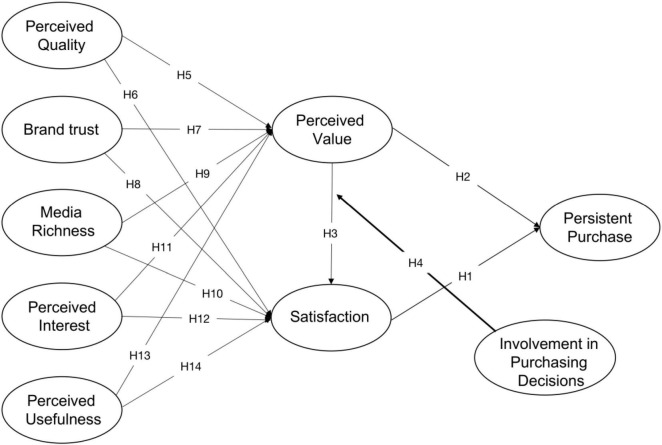
Concept model.

According to the theory above, after fully considering the characteristics of museums and drawing on the technical methods and research results of previous research, an integrative model is proposed in this research (Figure 1). Within the model there are 9 dimensions including PQ, BT, MR, PI, PU, PV, SAT, IPD, and PP, and puts forward 14 relevant hypotheses to discuss online consumers’ PP intention of museum cultural and creative products.

## Research Design and Methods

### Questionnaire Design

This research adopts questionnaires to understand the relationship among these dimensions. Combined with the characteristics of museum cultural and creative products, the measurement indicators used in this research were all set and optimized according to the questionnaire items in the current literature. A 7-point Likert scale was employed to measure the items (1 = strongly disagree, 7 = strongly agree). Aiming at ensuring the reliability of the research, before distributing the questionnaires, a pre-test was conducted on 100 respondents to modify the number, order and contents of questions based on their feedback, and the final questionnaires were distributed on a large scale after improvement.

In order to make sure that the respondents filled out the questionnaires based on their real experience and to ensure the authenticity and credibility, the first question of the questionnaire was set as “have you ever purchased museum cultural and creative products online?” If the answer was negative, the questionnaire was ended immediately. The first part of the questionnaire includes 19 items of 5 dimensions in the model, namely PQ, BT, PI, PU, and MR. The second part is about the purchasing characteristics of cultural and creative products, including 16 items based on PV, SAT, IPD, and PP. The last part is about the demographic characteristics of the tested ones such as gender, age, education level, monthly income, and other basic personal information. The contents of the questionnaire are shown in Table 1.

**TABLE 1:** Measurement scale.

Latent variable	Coding	Item	References
Perceived quality	PQ1	The quality and other product attributes of museum cultural and creative products sold online are in line with my expectations.	Beneke et al., 2013
	PQ2	The museum cultural and creative products sold online are consistent with the product descriptions on the website.	
	PQ3	The museum cultural and creative products sold online have quality assurance and can be returned or exchanged without reasons.	
	PQ4	Museum cultural and creative products sold online provide real and comprehensive information that can help me determine whether the product is what I need.	
Perceived interest	PI1	I think it is interesting to buy museum cultural and creative products online.	Mathwick et al., 2001
	PI2	I enjoy the process of buying museum cultural and creative products online.	
	PI3	Using museum cultural and creative products can bring me joy.	
	PI4	Museum cultural and creative products are very interesting.	
Brand trust	BT1	I have confidence in the brand of museum cultural and creative products.	Xu et al., 2021
	BT2	Museum cultural and creative product brands are always satisfying.	
	BT3	The brand of museum cultural and creative products can meet my expectations.	
	BT4	The brand of museum cultural and creative products reassures me and makes me feel no risk.	
Perceived usefulness	PU1	I was able to successfully buy museum cultural and creative products I wanted from the online store.	Wu et al., 2021
	PU2	The museum cultural and creative products purchased on the online store are in line with my expectations.	
	PU3	Museum cultural and creative products that are not easy to find in physical stores can be purchased through the online store.	
	PU4	I think the museum cultural and creative products in the online store are cheap and quality guaranteed.	
Media richness	MR1	The museum online store can provide instant feedback on my request.	Daft and Lengel, 1984; Levy and Gvili, 2015
	MR2	The museum online store presents product information in different formats (e.g., text, pictures, video, audio, animation, and 3D virtual environment).	
	MR3	The museum online store provides accurate cultural information of cultural and creative products through pictures and texts.	
Perceived value	PV1	I think the museum cultural and creative products purchased online are worth the money.	Li et al., 2007; Wang, 2007
	PV2	Using museum cultural and creative products can bring me more praise.	
	PV3	I think museum cultural and creative products are very innovative.	
	PV4	I think museum cultural and creative products can deepen my understanding of museum collections and contents.	
	PV5	Visiting the museum online store helps me get an impression of what I saw at the museum.	
Satisfaction	SAT1	I am generally satisfied with buying museum cultural and creative products online.	Zha and Wang, 2006
	SAT2	After purchasing museum cultural and creative products online, I started to like the way of online purchase.	
	SAT3	The experience of buying museum cultural and creative products online exceeds my pre-purchase expectations.	
	SAT4	Choosing to buy museum cultural and creative products online is a correct decision.	
Involvement in purchasing decisions	IPD1	Museum cultural and creative products are what I need.	Wu, 2015
	IPD2	I am very interested in museum cultural and creative products.	
	IPD3	Before buying a museum cultural and creative product, I read the relevant reviews and comments in detail.	
	IPD4	For museum cultural and creative products, I choose carefully when purchasing.	
Persistent purchase	PP1	I will continue to use online store to buy museum cultural and creative products.	He, 2008
	PP2	I will continue to use online store to buy museum cultural and creative products.	
	PP3	I will recommend to my friends to buy museum cultural and creative products online.	

### Sample and Procedure

The questionnaires were distributed online in November 2021, and 613 were collected by the deadline, of which 528 (86.1%) were considered valid. The questionnaires were screened for validity according to the following three principles: (1) the respondent need to complete all questions in the questionnaire, (2) the respondent should not choose the same option to answer all questions, and (3) the respondent must have purchased museum cultural and creative products online. In the final sample, 34.1% of respondents are male and 65.9% are female. In terms of age, the proportion of people under 36 is relatively balanced, they are under 25 (23.1%), 26–30 (29.7%), 31–35 (23.7%), 36–40 (14.6%), respondents over 51 accounted for the least (2.1%), followed by those aged 41–50 (6.8%). All the demographic information can be referred to in Table 2.

**TABLE 2:** Demographics of respondents (*N* = 528).

Variable	Group	Frequency	Percent
Gender	Male	180	34.1
	Female	348	65.9
Age	Under 25	122	23.1
	26–30	157	29.7
	31–35	125	23.7
	36–40	77	14.6
	41–50	36	6.8
	Over 51	11	2.1
Education level	High school and below	64	12.1
	College degree	148	28.0
	Bachelor degree	273	51.7
	Master’s degree and above	43	8.1
Monthly income	Less than 3000 RMB	29	5.5
	3001–5000 RMB	146	27.7
	5001–8000 RMB	157	29.7
	8001–15,000 RMB	118	22.3
	Over 15,000 RMB	78	14.8

### Data Collection and Analysis

SPSS 26, PROCESS 3.5 and Mplus 8.3 were adopted for statistical analysis of the data. Means and SDs were used to describe continuous variables, and frequencies and percents were used to describe categorical variables. Harman’s one-factor test was employed to test the common method biases, and Cronbach’s alpha was adopted as a reliability measure. Exploratory factor analysis and confirmatory factor analysis were resorted to explore the validity of the scale, and composite reliability (CR) and average variance extracted (AVE) to test the combined reliability and convergent validity, respectively. The correlation between variables was investigated by correlation analysis. If the square root value of AVE is greater than the correlation coefficient between the variable and other variables, it can be inferred that the variable is featured with good discriminant validity. The structural equation model of the latent variable was established through Mplus to explore the relationship between the variables in this research. Based on PROCESS of SPSS, Model 1 was used to explore the moderating effect of IPD on the relationship between PV and SAT. The moderating effect of IPD on the relationship among SAT, PV, and PP was explored through Model 7. The significant level is 0.05, that is, if *P* < 0.05, there is a statistically significant difference.

## Results

### Common Method Bias Test

The principal component analysis method was used to extract 9 components with initial eigenvalues greater than 1, and the CPV is 68.568%, therefore the principal component analysis method used in this research can better cover the main information. The unrotated CPV of the first common factor is 35.274%, which is less than 50% (Podsakoff et al., 2003), so it is considered that there is no serious common method bias in this research.

### Variable Description, Reliability, and Validity

#### Variable Description

The average values and SDs of each variable are shown in the table above. The average values of each variable in this research are between 5.039 and 5.938, that is, the respondents have relatively positive feedback and unified views.

#### Reliability Analysis

Cronbach’s alpha was adopted to conduct the measurement upon the reliability of the research model and its dimensions. Cronbach’s alpha is the most common method for internal consistency reliability test with value between 0 and 1. The higher the value, the more reliable it is. It is generally considered that the value over 0.7 has good reliability, and that greater than 0.9 has relatively ideal reliability. The analysis results of this research showed that the Cronbach’s alpha of each dimension is between 0.765 and 0.863, which are greater than 0.7 (Nunnally, 1967). Therefore, the reliability of each dimension in this research is considered good.

#### Validity Analysis

A confirmatory factor analysis model was constructed for each scale to analyze the results in Table 3: the AVEs of each variable in this research are between 0.517 and 0.620, which are greater than 0.5 (Fornell and Larcker, 1981), so they are considered to have good convergent validity. The CRs of each variable are between 0.768 and 0.873, higher than 0.70, so it is considered that each variable in this research has a good CR. Third, the fitting indexes of each scale are: χ^2^ = 735.573, *df* = 524, χ^2^/*df* = 1.404 < 5, RMSEA = 0.028 < 0.08, CFI = 0.976 > 0.9, TLI = 0.973 > 0.9, SRMR = 0.031 < 0.08, which all reach the standard, so the validity of the questionnaire is considered good from the perspective of confirmatory factor analysis.

**TABLE 3:** Variable description, reliability, and validity.

Variable	*M*	SD	Cronbach’s alpha	CR	AVE
1. Perceived quality (PQ)	5.329	0.815	0.823	0.828	0.549
2. Perceived interest (PI)	5.232	0.925	0.831	0.832	0.553
3. Brand trust (BT)	5.086	1.016	0.863	0.867	0.620
4. Perceived usefulness (PU)	5.532	0.898	0.841	0.842	0.572
5. Media richness (MR)	5.039	0.946	0.765	0.768	0.524
6. Perceived value (PV)	5.279	0.907	0.873	0.873	0.581
7. Satisfaction (SAT)	5.767	0.762	0.836	0.837	0.562
8. Involvement in purchasing decisions (IPD)	5.938	0.664	0.806	0.810	0.517
9. Persistent purchase (PP)	5.817	0.837	0.827	0.830	0.619

### Correlation Analysis and Discriminant Validity

Correlation analysis is to test the correlation between variables according to the correlation coefficient. Pearson’s correlation coefficient is commonly used in social science. If the correlation coefficient passes the significance test, it indicates that the correlation between the variables is either statistically positive or negative. If fails, the statistical correlation between the variables does not exist. When the correlation coefficient is <0.4, it indicates a weak correlation; when between 0.4 and 0.7, a moderately strong correlation; when >0.7, a strong correlation. In addition, if >0, a positive correlation; if <0, a negative correlation.

The correlation coefficients of the variables in this research are shown in Table 4, and the values on the diagonal line represent the AVE square root of this variable. In this research, there is a significant correlation between any two variables (*P* < 0.01), and Pearson’s correlation coefficients are between 0.326 and 0.587, that is, there is a moderate or weak positive correlation between the variables, which is in line with the hypothesis. The AVE square root of each variable is higher than its correlation coefficients with other variables, indicating that the variable is characterized with good discriminant validity, therefore the discriminant validity of each dimension in this research is considered good (Fornell and Larcker, 1981).

**TABLE 4:** Correlation analysis and discriminant validity.

	1	2	3	4	5	6	7	8	9
1. Perceived quality (PQ)	0.741								
2. Perceived interest (PI)	0.461**	0.744							
3. Brand trust (BT)	0.387**	0.440**	0.787						
4. Perceived usefulness (PU)	0.417**	0.473**	0.382**	0.756					
5. Media richness (MR)	0.343**	0.404**	0.326**	0.453**	0.724				
6. Perceived value (PV)	0.482**	0.494**	0.468**	0.506**	0.480**	0.762			
7. Satisfaction (SAT)	0.507**	0.552**	0.481**	0.529**	0.490**	0.587**	0.750		
8. Involvement in purchasing decisions (IPD)	0.373**	0.452**	0.400**	0.372**	0.378**	0.385**	0.454**	0.719	
9. Persistent purchase (PP)	0.526**	0.488**	0.389**	0.520**	0.438**	0.531**	0.548**	0.384**	0.787

**p < 0.01.

### Structural Equation Model Analysis

**FIGURE 2:**
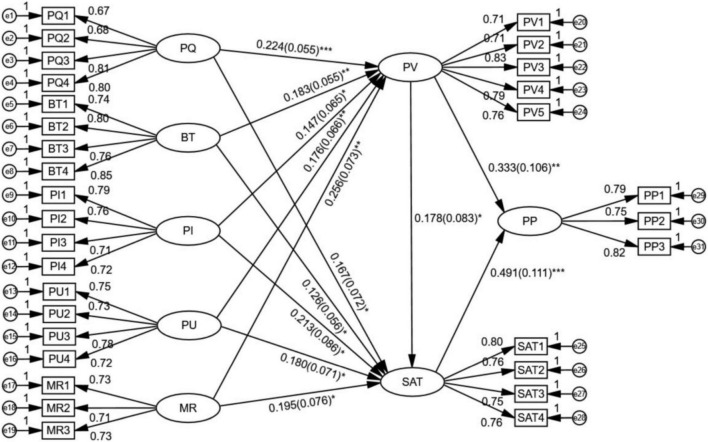
Results of structural equation model analysis. *p < 0.05, **p < 0.01, ***p < 0.001.

Mplus is adopted to establish structural equation model of latent variables to explore the impact of PQ, BT, PI, PU, and MR on PV and SAT, and the impact of PV and SAT on PP. The moderating effect of SAT between PV and PP is also analyzed. The results are shown in Figure 2.

The results of structural equation modeling (SEM) analysis are shown in Figure 1: the fitting indexes of structural equation model are: χ^2^ = 651.34, *df* = 411, χ^2^/*df* = 1.585 < 5, RMSEA = 0.033 < 0.08, CFI = 0.970 > 0.9, TLI = 0.967 > 0.9, SRMR = 0.038 < 0.08, which all meet the criteria of model fitting, so the model can be supported by the data and the model structure is considered good.

For the measurement model, the normalization coefficient (factor load) of each path is greater than 0.5, indicating that the factor load of the measurement model is relatively large and the structure of the measurement model in this research is good.

The impact of PQ, BT, PI, PU, and MR on PV: in terms of the impact of PQ on PV, *P* < 0.001, the discrepancy is statistically significant, β = 0.224, indicating that PQ significantly positively impacts on PV. Similarly, BT positively impacts on PV (*P* < 0.01, β = 0.183), PI significantly positively impacts on PQ (*P* < 0.05, β = 0.147), PU significantly positively affects PV (*P* < 0.01, β = 0.176), and MR significantly positively affects PV (*P* < 0.01, β = 0.256).

The impact of PV, PQ, BT, PI, PU, and MR on SAT: PV significantly positively impacts on SAT (*P* < 0.05, β = 0.178), PQ significantly positively impacts on SAT (*P* < 0.05, β = 0.167), BT significantly positively impacts on SAT (*P* < 0.05, β = 0.126), PI significantly positively impacts on SAT (*P* < 0.05, β = 0.213), PU significantly positively impacts on SAT (*P* < 0.05, β = 0.180), and MR significantly positively impacts on SAT (*P* < 0.05, β = 0.195).

The impact of PV and SAT on PP: PV significantly positively impacts on PP (*P* < 0.01, β = 0.333), and SAT significantly positively impacts on PP (*P* < 0.001, β = 0.491).

For the moderating effect of SAT between PV and PP, the Bootstrap method was used to estimate the confidence interval of the effect sizes. The number of iterations was set as 5000, and the confidence interval 95%. If 0 is not contained in the confidence interval, the effect is considered significant. The detailed results are shown in Table 5.

**TABLE 5:** Estimate of standardized effect sizes.

	Estimate	SE	95% LCI	95% UCI	Ratio
Total effect	0.390	0.101	0.214	0.585	–
Direct effect	0.302	0.113	0.073	0.494	77.4
Indirect effect	0.088	0.041	0.015	0.174	22.6

According to the results of Bootstrap test, the 95% confidence intervals of total effect, direct effect and indirect effect are [0.214, 0.585], [0.073, 0.494], and [0.015, 0.174], respectively, all excluding 0. The total effect, direct effect and indirect effect are all considered significant with the effect sizes as 0.390, 0.303, and 0.088, and the indirect effect accounts for 22.6% of the total effect. Therefore, it is believed that SAT partially moderates PV and PP.

### Moderating Effect of Involvement in Purchasing Decisions on the Relationship Between Perceived Value and Satisfaction

**FIGURE 3:**
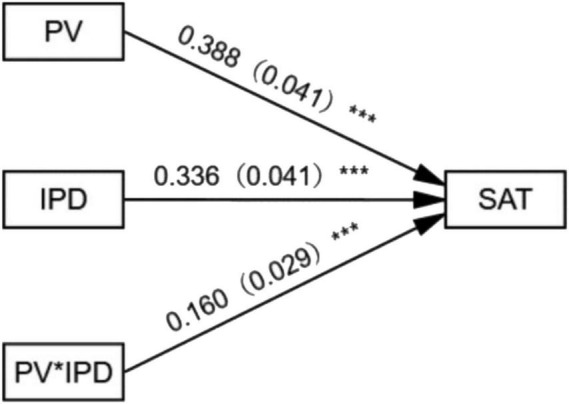
Moderating effect of involvement in purchasing decisions on the relationship between perceived value and satisfaction. ***p < 0.001.

For the moderating effect of IPD on the relationship between PV and SAT, Process of SPSS was used to set gender, age, education level, and monthly income as control variable, PV as independent variable, IPD as moderator variable, and SAT as outcome variable to present the main path. The results are shown in Figure 3.

The impact of IPD on PV and SAT: the model fitting index *R* = 0.676, *R*^2^ = 0.457, *F* = 62.621, *P* < 0.001, so the model passes the significance test and is supported by data (Fang et al., 2015). The results of regression analysis show that the moderator variable, IPD significantly positively impacts on SAT (*P* < 0.001, *B* = 0.336). In terms of the moderating effect, the interaction item, perceived value × involvement in purchasing decisions (PV × IPD) significantly positively impacts on SAT (*P* < 0.001, *B* = 0.160), so it is believed that IPD positively moderates the relationship between PV and SAT.

**FIGURE 4:**
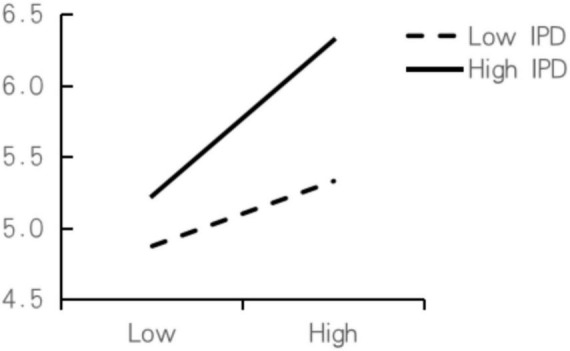
Moderating effect of involvement in purchasing decisions on the relationship between perceived value and satisfaction.

A decomposition diagram of the moderating effect is drawn based on the regression coefficient (Figure 4). Under the condition of high-degree IPD, the impact of PV on SAT (seen as the slope) is greater than that under the condition of low-degree IPD. Therefore, it is believed that IPD positively moderates the relationship between PV and SAT.

### The Moderating Effect of Involvement in Purchasing Decisions on the Mediating Role of Satisfaction Between Perceived Value and Persistent Purchase

Based on the aforementioned research results, PROCESS Model 7 of SPSS was employed to test the moderating effect of IPD on the mediating role of SAT between PV and PP. Gender, age, education level, and monthly income were included as control variable, PV as independent variable, SAT as mediating variable, IPD as moderating variable, and PP as outcome variable to estimate the moderated mediating effect. The results are shown in Table 6.

**TABLE 6:** Moderated mediating effect.

	Effect	SE	95% LCI	95% UCI
Low (−SD)	0.108	0.026	0.059	0.162
Middle (0)	0.149	0.028	0.096	0.207
High (+SD)	0.189	0.034	0.123	0.259
Moderated mediation	0.061	0.017	0.032	0.098

When the moderating variable is reduced by 1 SD from the mean, the moderated mediating effect size is 0.108, and the 95% confidence interval is [0.059, 0.162] with 0 excluded. When the moderating variable is 1 SD higher, the moderated mediating effect size is 0.189, and the 95% confidence interval is [−0.123, −0.259] with 0 excluded. The moderated mediation is 0.061, and the confidence interval is [0.032, 0.098] with 0 excluded, so the moderated mediating effect is considered to be significant (Wen et al., 2006).

## Results and Discussion

Several key findings emerged from the validation of the SEM and various validation results, which are discussed in this section.

H1 is valid, indicating that the consumer SAT of shopping on the online museum platform positively impacts on their PP, and the path coefficient is the highest. That is to say, SAT is the most important factor in determining whether consumers will continue to purchase museum cultural and creative products online. Satisfied buyers are more likely to generate trust in the future, numerous research and studies have confirmed the effect of SAT on PP. H2 is valid, indicating that the consumer PV of online museum stores positively impacts on PP. The path coefficient is second only to H1, and the positive impact of PV on PP is stronger than that on SAT, indicating that PV plays an important role in marketing strategy management. H3 is valid, indicating that consumer PV positively affects their SAT with online purchases of museum cultural and creative products, confirming that PV has a particularly significant impact on SAT and purchase intention. Meanwhile, through the Bootstrap test, this research found that SAT partially mediates the PV and PP. Therefore, in the design and marketing of cultural and creative products, PV and SAT play an important role, which emphasizes the urgent need to increase PV and SAT in a positive direction in order to boost consumers’ PP. According to the results of this research, such positive factors can be categorized into consumers’ PQ of museum cultural and creative products, BT in museums, MR perception of online museum stores, PI of museum cultural and creative products and online purchases and PU of museum cultural and creative products and online platforms, which will be discussed in detail.

H5 and H6 are valid, indicating that PQ positively impacts on the PV and SAT of online museum store consumers. H7 and H8 are valid, indicating that BT also positively impacts on the PV and SAT of online museum store consumers. Strong BT will strengthen customers’ loyalty to the brand, and they are more likely to choose this brand when they purchase. Therefore, brand power is an important part of winning. Building a communication platform between customers and brand is extremely important to intensify customer trust. Only through building brand consistency and systemicity, can BT last. H9 and H10 are valid, indicating that MR positively impacts on the PV and SAT of online museum store consumers. H11 and H12 are valid, indicating that PI positively impacts on the PV and SAT of online museum store consumers. Interesting product design can strengthen consumers’ cognition of products and stimulate their emotions. H13 and H14 are valid, indicating that PU positively impacts on the PV and SAT of online museum store consumers. The impact of PU on PV can offer new breakthroughs for the marketing of museum cultural and creative products, and ultimately leads to the realization of market value.

H5 and H13 present greater path coefficients, indicating that PU and PQ are important factors of consumers’ PV, and PU will increase consumers’ willingness to purchase products, which verifies the research of Beneke et al. (2013) and Ji (2015). It also means that the development and design of museum cultural and creative products need to focus on consumer demand and product quality. In addition to aesthetic value, it must also combine the functional value of cultural products, integrate creativity and life, and design daily necessities with collection value. Then it comes to H10 and H12: MR and PI positively impact on SAT, which is consistent with the hypotheses, validating that MR and PI are necessary variables to effectively predict consumers’ PU. If the provider of a product or service increases its interest, consumers are likely to pay more attention to such product or service and increase their willingness to purchase or use it. Consumers can feel the historical connotation through museum cultural and creative products and obtain the information and knowledge they want. Therefore, the development of personalized and interesting cultural and creative products and the improvement of the interactive interest of museums’ online platform will positively affect consumers’ subjective experience perception, thereby enhancing consumers’ PV of museum cultural and creative products, and ultimately stimulating their PP.

H4 is valid, indicating that consumers’ IPD positively moderates the impact of PV on SAT. Meanwhile, the results of the moderating effect test on the mediating effect of IPD among SAT, PV, and PP showed significant moderated mediating effect. It proves that consumers’ attention to a specific museum cultural and creative product has an important impact on their behavior before purchasing decisions. When the information about the product or service is more comprehensive before consumers’ purchase behavior, the improvement of consumer SAT can be enhanced and ultimately affect the formation of purchasing decisions (Browne and Kaldenberg, 1997). The IPD on museum cultural and creative products is mainly reflected in the depth of thinking about culture and history brought about by the products, users’ interest stimulated in museum cultural and creative products, and the possibility to view relevant evaluations in detail. Based the above, the SAT brought by triggering users to think about the depth of culture and history and the concentration and devotion brought by interest can improve consumers’ SAT with museum cultural and creative products, and thus affect their purchasing intentions. This is a chain reaction formed from the migration of interest points driven by products and services themselves. If consumers can be fully activated in information acquisition and interest stimulation, their consumption behavior can be positively influenced. Therefore, as an online platform for museum cultural and creative products, it is necessary to focus on ways to increase the degree of IPD. In terms of information involvement of online stores and digital museum platforms, MR and PI are important factors for displaying cultural and creative products, which can improve users’ SAT and ultimately affect users’ purchasing intentions. Museum cultural and creative products have obtained longer-term opportunities of publicity and display through online platforms and more selling opportunities through online marketing. To this end, relying on the data and key information obtained from online channels, effectively coordinating the focuses of product development, and disclosing the biased information in the layout of online marketing can build a more stable and sustainable platform for the online marketing and industrial development of museum cultural and creative products.

## Research Conclusions and Recommendations

### Theoretical Significance

The most significant contribution of this research lies in the establishment of the model of influencing factors of online consumers’ PP of museum cultural and creative products from multiple dimensions such as PQ, BT, MR, PI, PU, PV, and IPD. In addition, all the above dimensions directly or indirectly impact on online consumers’ PP. The results show that SAT comes from the collective effect of PQ, BT, MR, PI, and PU, and SAT plays a mediating role between the PV of online museum store users and their PP. Among the influencing factors of PV, PU, and PQ play a major role, followed by BT, PI, and MR. Among the influencing factors of SAT, MR is the most influential one, followed by PU, PI, PV, PQ, and BT.

### Practical Implications

The results of this research can help practitioners of online museum operating and cultural and creative product development to provide better products and services based on the influencing factors of online consumers’ PP of museum cultural and creative products. According to the conclusions above, recommendations are put forward as follows.

1. For museum brand operation: (1) strengthen the brand operation of cultural and creative products to establish a distinctive and prominent position and unique museum images in the minds of consumers, form a differentiated advantage and enhance the brand added value; (2) integrate advanced information technology in the sales of museum cultural and creative products, enhance the experiential richness and entertainment of products, and further promote the achievement of successful purchase; (3) strictly control the distribution channels to ensure that consumers receive authentic and quality-assured cultural and creative products through online platforms; (4) in the online sales and operation of museum cultural and creative products, intensify the construction of information to meet users’ cognitive needs, work hard in the presentation of contents, attach importance to multiple information communication methods such as innovative communication methods on digital platforms, optimize visual communication effects, create intuitive interfaces of product details, provide real and comprehensive information, optimize website design elements including color matching and typography, improve the aesthetic value of websites, and emphasize the cultural and historical connotation and functional attributes of the products; (5) integrate online and offline interactive methods or set up games and other multimedia methods to display product types and culture based on the product characteristics to enhance the interest and enrich the cultural attributes of museum cultural and creative products.

2. For the development and design of museum cultural and creative products: (1) focus on the integration of cultural values and the function of continuity, design products to popularize historical and cultural knowledge, and meet the increasing “consumer-oriented” demands for cultural consumption; (2) pay attention to the quality and functionality of the products, integrate culture and creativity with daily needs, and design products with collection value and practicality; (3) pay attention to the interesting design of museum cultural and creative products and consumers’ behavior and reflection in the process of using products, for highly comfortable products and those that can arouse users’ memory of cultural relics or help them recall fragments in the past will improve consumers’ PV and stimulate PP; (4) pay attention to the participation of consumers in the design and development stage of museum cultural and creative products, emphasize the development of digital products featured with interaction experience through panoramic or VR technology, obtain market information in a timely and effective manner, and transform market information into selling points or value points of cultural and creative products, so as to guide the market to the design and development stage of such products.

### Limitations and Future Research

The following limitations of this research may indicate some research directions in the future, which can be continuously improved and deepened in the follow-up research and studies. First of all, this research is not focused on a certain museum or several museums, nor their cultural and creative products, so it cannot verify whether the macro-level research results are biased in the implementation of specific issues. In future research, one or several museums and their cultural and creative products with specific attributes can be deeply analyzed according to different research stages to obtain more in-depth research models and results. Secondly, this research only focuses on modeling and consumers’ internal perception from a macro perspective, and has not conducted specific analysis toward different online platforms, such as the perceived differences of museum flagship stores across different media including traditional e-commerce, short video and live stream platforms, cloud exhibitions of museums, and virtual reality. Last but not least, this research adopts SEM as the research method, and qualitative research can be attached in the future to complement effects which cannot be explained by quantitative data to a deeper degree.

## Data Availability Statement

The original contributions presented in the study are included in the article/supplementary material, further inquiries can be directed to the corresponding author.

## Ethics Statement

Ethical review and approval was not required for the study on human participants in accordance with the local legislation and institutional requirements. Participants provided written informed consent to participate in the study.

## Author Contributions

ML and ZM: conceptualization and data collecting. ML and CL: methodology and investigation. ML: writing – original draft preparation. All authors have read and agreed to the final version of the manuscript.

## Conflict of Interest

The authors declare that the research was conducted in the absence of any commercial or financial relationships that could be construed as a potential conflict of interest.

## Publisher’s Note

All claims expressed in this article are solely those of the authors and do not necessarily represent those of their affiliated organizations, or those of the publisher, the editors and the reviewers. Any product that may be evaluated in this article, or claim that may be made by its manufacturer, is not guaranteed or endorsed by the publisher.
